# Expression pattern of three-finger toxin and phospholipase A_2 _genes in the venom glands of two sea snakes, *Lapemis curtus *and *Acalyptophis peronii*: comparison of evolution of these toxins in land snakes, sea kraits and sea snakes

**DOI:** 10.1186/1471-2148-7-175

**Published:** 2007-09-27

**Authors:** Susanta Pahari, David Bickford, Bryan G Fry, R Manjunatha Kini

**Affiliations:** 1 Protein Science and Conservation Ecology Laboratories, Department of Biological Sciences, National University of Singapore, 117543, Singapore; 2 Department of Biochemistry & Molecular Biology, Bio21 Institute, University of Melbourne, Parkville, Victoria, 3010 Australia; 3 Deparment of Biochemistry, Medical college of Virginia, Virginia Commonwealth University, Richmond, VA 23298-0614 USA; 4 Center for Post Graduate Studies, Sri Bhagawan Mahaveer Jain College, 18/3, 9^th ^Main, Jayanagar 3^rd ^Block, Bangalore, India

## Abstract

**Background:**

Snake venom composition varies widely both among closely related species and within the same species, based on ecological variables. In terrestrial snakes, such variation has been proposed to be due to snakes' diet. Land snakes target various prey species including insects (arthropods), lizards (reptiles), frogs and toads (amphibians), birds (aves), and rodents (mammals), whereas sea snakes target a single vertebrate class (fishes) and often specialize on specific types of fish. It is therefore interesting to examine the evolution of toxins in sea snake venoms compared to that of land snakes.

**Results:**

Here we describe the expression of toxin genes in the venom glands of two sea snakes, *Lapemis curtus *(Spine-bellied Sea Snake) and *Acalyptophis peronii *(Horned Sea Snake), two members of a large adaptive radiation which occupy very different ecological niches. We constructed cDNA libraries from their venom glands and sequenced 214 and 192 clones, respectively. Our data show that despite their explosive evolutionary radiation, there is very little variability in the three-finger toxin (3FTx) as well as the phospholipase A_2 _(PLA_2_) enzymes, the two main constituents of *Lapemis curtus *and *Acalyptophis peronii *venom. To understand the evolutionary trends among land snakes, sea snakes and sea kraits, pairwise genetic distances (intraspecific and interspecific) of 3FTx and PLA_2 _sequences were calculated. Results show that these proteins appear to be highly conserved in sea snakes in contrast to land snakes or sea kraits, despite their extremely divergent and adaptive ecological radiation.

**Conclusion:**

Based on these results, we suggest that streamlining in habitat and diet in sea snakes has possibly kept their toxin genes conserved, suggesting the idea that prey composition and diet breadth may contribute to the diversity and evolution of venom components.

## Background

The composition of snake venoms varies widely both within a species and among closely related species [1-4]. This variation is proposed to be due to changes in the diet of snakes, based on the findings in the variation of intraspecific venom composition in a pit viper, *Calloselasma rhodostoma*, a land snake [2]. Land snakes depend on a diversity of prey including lizards (reptiles), frogs and toads (amphibians), birds (aves), insects (arthropods), and rodents (mammals)[5,6]. They probably require a range of toxins that target different groups of prey species since there is variation in venom's ability for immobilization and killing across such a variety of prey. Toxins which are used for systematic prey envenomation found to have several isoforms in their venom gland as evident from global cataloguing of snakes toxin gene expression [3,7-16] and it has been correlated that variation in prey favors the evolution of multiple isoforms of toxins in venoms [9,17]. The variety of isoforms is believed to have been achieved through frequent gene duplications accompanied by an accelerated rate of evolution [18-20] similar to the generation of adaptive response in immunoglobulins and major histocompatibility complex genes in response to a wide range of foreign antigens [21]. Thus, a birth-and-death mode of evolution generates diversity in toxins allowing snakes to feed on a variety of prey species [22].

Elapid snakes are a monophyletic clade of approximately 300 species in 61 genera [23]. True sea snakes (Hydrophiinae) and sea kraits (*Laticauda *spp.) form two elapid clades that have evolved independently but are either rooted within (true sea snakes) or basal to (sea kraits) the terrestrial Australo-Papuan elapids rather than other elapid groups [24-28]. These snakes have adapted to marine life and undergone many changes in foraging behavior, morphology and diet [29]. As a result, although their feeding systems are confined to prey of a single vertebrate class (fishes), they often specialize on particular types or families of fish [30,31]. With such restrictions in both diet and habitat, one might expect low diversity in toxin components (relative to snakes with broader diets), as has been shown to be the case in the hydrophiinae subfamily [9]. We showed by analyzing the cDNA library of *Aipysurus eydouxii *that its 3FTx gene is inactivated by a dinucleotide (TT) deletion [32] and the evolution of its PLA_2 _isoenzymes, unlike those from other snake venoms, is decelerated [33]. As this unique sea snake feeds exclusively fish eggs [31], we suggested that a shift in the diet of *A. eydouxii *may have resulted in the relaxation of selection pressures on its 3FTx and PLA_2 _genes

Here, we examined the total gene expression pattern of two other sea snakes, *Lapemis curtus *and *Acalyptophis peronii*, which have distinct and different habitats and feeding systems. *L. curtus *inhabits many different areas like open sea, estuaries, and coral reefs, whereas *A. peronii *inhabits only sandy areas between coral reefs [34]. *L. curtus *in contrast to other sea snakes is a generalist feeder and its diet is one of the most diverse of all sea snakes [30,31,34-36]. Its prey consists of fishes (90%; 31 different families) and very few invertebrates (10%; squid and cuttlefish) [30,35,36]. Additionally, *L. curtus *cohabits with other sea snakes, and consequently may be overlapping in diet. In contrast, the diet of *A. peronii *is confined mainly to gobies (one class of sea fish) [34] and it is a diet and habitat specialist. Because these two snakes are members of a large adaptive radiation of the *Hydrophis *lineage and they might have diverged very rapidly, differences in their venoms might also be widely divergent if they track diet specialization. On the other hand, if diet specialization within a constrained group of prey (e.g., only fish), drives more of a streamlining of venom evolution, then we might expect there to be few or no changes in venom constituents. Therefore, it could be interesting to compare the total toxin gene expression of these two sea snakes.

We constructed cDNA libraries of the venom glands from *A. peronii *and *L. curtus *specimens and sequenced about 200 clones of each. Sampling of transcriptoms indicates the presence of any new and/or rare families of toxins and enables analyses of the molecular evolutionary trends among toxin genes. Further, to compare the evolution of toxin genes among land snakes, sea snakes, and sea kraits, we calculated the evolutionary distances using all available sequences of two principle components of the toxin proteome, 3FTx and PLA_2_.

## Results

### cDNA libraries of *Lapemis curtus *and *Acalyptophis peronii *venom glands

**Figure 1:**
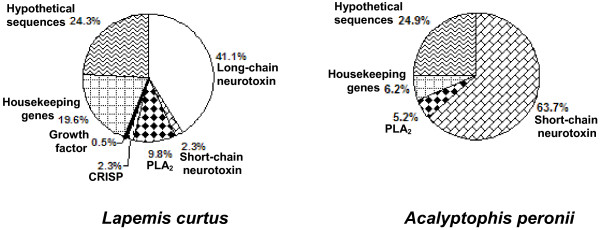
Distribution of transcripts in the venom glands of *Lapemis curtus *and *Acalyptophis peronii*.

We obtained 4 and 5 μg of mRNA from 30 mg of venom gland tissues of *Lapemis curtus *and *Acalyptophis peronii*, respectively. We constructed two separate cDNA libraries using 1 μg of mRNA from each preparation. From the clones containing inserts, we randomly selected 250 and 225 clones, respectively. From these clones we were able to obtain sequences of 214 cDNA clones from *L. curtus *and 192 cDNA clones for *A. peronii*. Figure 1 shows the distribution of clones in both venom glands.

### *Lapemis curtus *library

#### 3FTx

To date, three long-chain isoforms of 3FTx (AAL54893, AAL54892 and ABN54806) and four short-chain isoforms of 3FTx (AAL54894, AAL54895, P68416 and ABN54805) [37]have been reported from *L. curtus *venom. We found cDNA clones encoding both long-chain isoforms (AAL54893 and AAL54892) of 3FTx in the library (41% abundance, Figure 1) and the ratio between the number of clones of isoforms AAL54893 and AAL54892 was ~10:1. We also found cDNA clones encoding a short-chain 3FTx (AAL54894; ~2% abundance, Figure 1) [37]. No variation was observed in the coding sequence of the mature proteins with AAL54893, AAL54892 and AAL54894. However, we could not detect the long chain isoform (ABN54806) and three other short-chain isoforms of 3FTx (AAL54895, P68416 and ABN54805) [37,38].

#### PLA_2_

So far, three isoforms of PLA_2 _of *L. curtus *(AAL55556, AAL55555 and AAL54920) have been reported [39]. We were only able to detect one isoform which is completely identical at the nucleotide level with AAL55555 (~10% abundance, Figure 1).

#### CRISP

Partial sequences of two isoforms (Q8UW25 and Q8UW11) of CRISP from *L. curtus *venom glands have recently been reported. Our cDNA library contained ~2% clones coding for Q8UW25 isoform without any variation at nucleotide level (Figure 1).

#### Others

The cDNA library has a singleton presence of a growth factor (AY742212) which shows significant identity to Platelet Derived Growth Factor (PDGF). The partial sequence shows 70% identity to the C-terminus of the predicted PDGF-D isoform from *Gallus gallus *(chicken). Although growth factors such as NGF [40] and VEGF [41] are known to be present in the venom, this is the first report of PDGF-like protein sequence from the venom gland. However, further studies are needed to confirm the presence of PDGF protein in the venom.

*L. curtus *library contained ~20% housekeeping genes (Figure 1), including ribosomal RNA, ribosomal proteins and cytochromes. In addition, ~25% of cDNA sequences did not show significant identity to toxins or metabolic genes (Figure 1). BlastX search of these sequences showed poor or only partial identity to any protein sequences with other organisms or no match at all. These unknown sequences in most of the cases are partial, singleton clones. However, their origin (venom gland or marginal contamination of surrounding tissues) still needs to be established.

### *Acalyptophis peronii *library

#### 3FTx

Amino acid sequences of two isoforms of short-chain 3FTx have been reported earlier [42,43]. Gln43 of the major isoform (AY742211) has changed to Glu43 in the minor isoform (AY742210) [43]. In *Acalyptophis peronii *library, the short-chain 3FTx was most abundant (~64%) (Figure 1) and there are two isoforms of 3FTx in equal numbers (60 and 62 respectively). These two isoforms (AY742210 and AY742211) have three nucleotide changes in their signal sequences leading to substitution of Thr7 (ACC) and Leu8 (TTG) by Ser7 (TCC) and Pro8 (CCG), respectively. However, no variation was observed in the coding sequence of the mature protein and the deduced protein sequence corresponds only to the major isoform [42]. As we did not obtain clones corresponding to the minor form, we propose that the minor form is most likely due to deamidation of Gln43 [44] and not a separate gene product. Generally in toxin families, it has been observed that the signal peptide regions, 5'UTR and 3'UTR are highly conserved, whereas the mature protein region shows a number of substitutions [19,45]. In contrast, the two isoforms of short-chain 3FTx differ in their signal peptide region but not in the mature protein in this case. It would be interesting to examine the importance of these substitutions.

#### PLA_2_

So far no PLA_2 _sequences from *A. peronii *have been reported. We found partial clones having 3' terminal sequences of PLA_2 _in *A. peronii *library (~5%; Figure 1). They show 100% identity in the 3'UTR region with *L. curtus *PLA_2 _(AAL55556 and AAL54920). Further identification and characterization of full length PLA_2 _is underway.

#### Others

The cDNA library contains ~6% clones encoding housekeeping genes (Figure 1). These include NADH dehydrogenase, ribosomal proteins and Ca^2+ ^binding proteins (calglandulin). The latter class of protein has been implicated in toxin secretion [46,47]. Like the *L. curtus *library, the *A. peronii *library also contained ~25% with no homology to any known toxin or housekeeping genes (Figure 1). As earlier, in most cases these sequences are partial, singleton clones and their origin needs to be verified.

### Intra and interspecific relationship of 3FTx and PLA_2 _sequences

The number of available protein sequences encoding 3FTx and PLA_2 _were higher than cDNA sequences because most of the sequences have been reported from direct protein sequencing. Therefore, we used protein sequences to calculate intra and interspecific pairwise distances for land snakes, sea snakes and sea kraits. It should be noted that due to paucity of the available data the number of species and number of short-chain 3FTx used for the calculations for land snakes, sea snakes and sea kraits were not the same.

**Figure 2:**
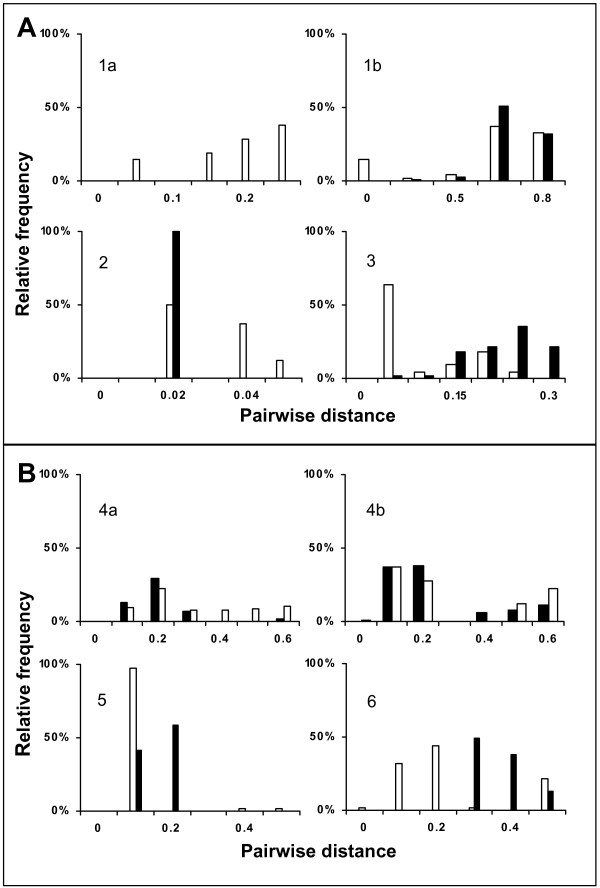
Pairwise intraspecific (white bar) and interspecific (black bar) distances for land snakes, sea snakes and sea kraits. Panel A: 3FTx (1a and 1b: land snakes; *Pseudonaja textilis and Bungarus *species respectively), 2 and 3: sea kraits and sea snakes respectively. Panel B: PLA_2_: (4a and 4b: land snakes; Australian elapids and *Bungarus *species respectively), 5 and 6: sea kraits and sea snakes respectively. RF denotes relative frequency.

For short-chain 3FTx, 37% of the intraspecific distances of both *Pseudonaja textilis *and *Bungarus *species (land snakes) are in the range of (0.2–0.3) and (0.7–0.8) respectively, while 63% of the intraspecific distances of sea kraits fall in the range of (0.1–0.2), and most of the intraspecific pairwise distances of sea snakes are in the range of (0.02–0.04) (Figure 2A). Interspecific pairwise distances also appear higher (50% in the range of 0.7 for *Bungarus *species) for land snakes, and lower for sea snakes (100% in the range of 0.02). Interspecific distances of 3FTx for *Pseudonaja *species were not calculated because sequences were only available from one species (*P. textilis*). The higher genetic distances of 3FTx in land snakes indicate higher levels of genetic diversity compared to sea snakes, where sequences were much more conserved. The genetic diversity within sea kraits is intermediate in both intra and interspecific comparisons. For PLA_2_, 22% of the Australian elapids and 36% of the *Bungarus *species intraspecific distances fall between (0.1–0.3) and (0.1–0.2) respectively. On the other hand, 97% and 44% of the sea snakes' and sea kraits' intraspecific distances ranged from (0.1–0.2) and (0.2–0.3) respectively (Figure 2B). Interspecific distances of PLA_2 _for Australian elapids, *Bungarus *species and sea kraits and sea snakes have comparable values (30%–60% in the range of 0.2–0.3; Figure 2B). But in sea snakes, interspecific distances (58% fall between 0.2–0.3) appear lower than the intraspecific distances. One of the possibilities for this reverse trend can be due to poor phylogenetic resolution among species in the hydrophiinae subfamily [48,49]

## Discussion

Snake venoms are a rich and diverse source of pharmacologically active proteins and peptide components [50,51]. Some of these components are enzymes, whereas others are nonenzymatic proteins or polypeptides. Most of these components are offensive weapons to capture the prey, injection of venom into prey leads to immobilization, death and can subsequently aid in digestion as well [52,53]. Venom might also be used for defensive purposes to keep possible predators away. Venom systems appear to have evolved to meet some of these goals, a single time in reptile evolution, at the base of the Toxicofera [54,55].

In this work, we show the high abundance of 3FTx in the venoms of sea snakes (41% for *Lapemis curtus *and ~64% for *Acalyptophis peronii*) while PLA_2 _is a distant second largest group (~10% for *L. curtus *and ~5% for *A. peronii*) of sea snake toxins. Overall, both the 3FTx and PLA_2 _do not possess an abundance of different isoforms generating significant variation in the venom composition. The fact that we did not detect some of the isoforms of these two groups of toxins as previously reported in *L. curtus *may be either due to regional variation within the species or a sampling artifact since the cDNA library was generated from venom glands of a single snake. However, both groups of toxins appears to be simple and do not have noteworthy diversity in their isoform compositions. It suggests that sea snake venoms genes are quite conserved, and therefore lack the diversity in its venom composition as observed for land snake and sea kraits. However, additional data from gene expression profile, frequency of gene duplication and accelerated evolution profile of sea snakes is needed to further test this hypothesis.

Comparison of intraspecific distances among 3FTx showed that the maximum value for land snakes is 0.8 whereas sea snakes are at 0.03 and sea kraits, 0.2 (Figure 2A). The variation between land and sea snakes is about 30 fold, whereas land snake and sea krait differ only 4 fold. However, this level of variation has not been found in PLA_2 _genes. In land snakes, the maximum intraspecific distance is 0.2 for land snakes and sea kraits, whereas sea snakes have a maximum value of 0.1, indicating a difference of only 2 fold (Figure 2B). Interspecific distances, for both 3FTx and PLA_2_, on the other hand, show greater or equal values than the intraspecific differences in land snakes and sea kraits (Figure 2A and 2B). From the genetic distance data, it is obvious that 3FTx is gaining more variability than PLA_2_. This is probably relevant because envenomation by elapid snakes is usually characterized by rapid neurotoxic complications due to presence of large amounts of neurotoxins [56]. Overall, our calculation for the intra and interspecific variation in both 3FTx and PLA_2 _appears distinct among land snakes, sea snakes and sea kraits indicating the probable existence of distinct evolutionary patterns that separate these groups.

Interestingly, the conservation of toxin diversity in sea snakes is not confined within species, it extends across different genera. For example, *Enhydrina schistosa*, a common sea snake, has just two neurotoxins (P25492 and P25493) [57]. The toxin P25492 is identical in sequence to a short-chain neurotoxin found in *Lapemis curtus *venom [38] and the other toxin, P25493, is identical to the short-chain neurotoxins found in venoms of *Hydrophis cyanocinctus *[58] and *Pelamis platurus *[59]. In contrast, among 276 3FTxs reported to date [22], we could not find a single 3FTx common across different genera of land snakes. Conservation of toxin sequences, even across genera of marine snakes is possibly due to a highly constrained niche, and the streamlined nature of their venoms is responsible for the remarkable degree of antivenom cross-reactivity [60].

The analysis of our cDNA libraries indicated that the *Lapemis curtus *venom is marginally more diverse than that of *Acalyptophis peronii*. The *L. curtus *library contains CRISP and growth factor isoforms in addition to 3Ftx neurotoxins and PLA_2 _enzymes. Chen et al. (AAV98367) reported the presence of a kallikrein toxin in *Lapemis curtus *venom as well. Recruitment of additional toxin families like CRISP, growth factor, kallikrein toxin may be due to its broad dietary requirements. In contrast, *A. peronii *venom glands contain only neurotoxins and PLA_2 _in high concentration and ittargets only gobies as its diet [30,31,34-36]. Therefore, evolution of toxin(s) in a generalist (*L. curtus*) and a restricted feeder (*A. peronii*) appear to be different. This does not indicate that other toxin classes are not expressed at low levels; more rigorous sequencing may reveal rarer transcripts.

The toxin expression profile data from cDNA library of *L. curtus *and *A. peronii *and a relationship between their habitat and diet may suggest that ecological variables presumably played a major role in determining the trajectory of their evolutionary paths along ecological niches (specialist and generalist) and not completely because of a distant phylogenetic relationship between them. There are however, a few specific cases available in the literature, where a relationship between intraspecific variations in venom with respect to dietary preferences has not been found [61, 62, 63]. Do these specific exceptions prove the general rule, or is there a threshold where the evolution of toxins becomes decoupled from feeding ecology and/or diet? These questions remain cogent for the future of toxin evolution research and we propose that sea snakes will remain major players in helping to understand how toxin evolution and feeding ecology are linked.

## Conclusion

Global cataloguing of toxin expression shows conserved expression pattern of two main families of toxins, 3FTx and PLA_2_, in two sea snakes venom giving rise to a simple venom composition relative to land snakes and sea kraits. Genetic distance values of 3FTx and PLA_2 _toxins show a more diverse trend of evolution for land snakes and sea kraits than to sea snakes. As the diet breadth (prey items) expands from sea snakes to land snakes (sea kraits as intermediate), we suggest that these trends in evolution of toxins may be linked to their diet.

## Methods

### Collection of venom glands

*Lapemis hardwickii *has been synonymized with *Lapemis curtus * [64] so *L. curtus *is used in this paper. One specimen of *L. curtus *and another of *A. peronii *were collected from Albatross Bay in Weipa, Queensland, Australia. Venom glands were dissected from each of these freshly caught snakes. Two glands from each snake were used for the construction of cDNA libraries. Although sample sizes are small for each species, the difficulty in acquiring specimens or keeping individuals in captivity make even these small sample sizes extremely valuable and worthy of study.

### Library construction, sequencing and analysis

Total RNA was extracted from the venom glands using RNeasy Mini Kit (Qiagen, Hilden, Germany). The integrity of total RNA was checked by agarose gel electrophoresis. The mRNA was purified using mRNA isolation kit (Roche Applied Science, Mannheim, Germany). The purified total mRNA was used to make the cDNA library following the instructions of the SMART cDNA library construction kit (Clontech, Mountain view, California, USA). The library was packaged using Gigapack gold packaging extract (Stratagene, Cedar Creek, Texas, USA). Individual clones were rescued from randomly selected white plaques and grown in (Luria broth + ampicillin) medium. Plasmids were purified using QIAprep spin miniprep kit (Qiagen, Hilden, Germany). Purified plasmids were sequenced by cycle sequencing reaction using the BigDye Terminator v3.1 kit (Applied Biosystem, Foster City, California, USA) and with an automated DNA sequencer (Model 3100A, Applied Biosystem, Foster City, California, USA). Sequences were compared to cDNA and protein sequences in NCBI database using BLAST program (BlastN and BlastX) and identical (or similar) clones were clustered. Each cluster was aligned using the program ClustalW in European Bioinformatics Institute site.

### Calculation of genetic distances

Genetic distances were compared by calculating intra and interspecific pairwise distances for the 3FTx and the PLA_2 _enzymes. All available protein sequences of 3FTx (short-chain isoforms) and PLA_2 _of land snakes, sea kraits *and *sea snakes were retrieved [see additional file 1]. Redundant sequences and signal peptides were removed and aligned. Aligned sequences were analyzed in PAUP* version 4.0 program [65] using the pairwise distance algorithm (uncorrected distances, kimura-2 parameters) for both within and between species. The pairwise distances were then plotted as a group for land snakes, sea snakes and sea kraits.

## Accession numbers

Nucleotide sequence data reported here have been deposited in GenBank under accession numbers [GenBank: AY742212, GenBank: AY742210, GenBank: AY742211].

## Competing interests

The author(s) declares that there are no competing interests.

## Authors' contributions

SP has performed the experiments, data analysis, writing and extension of the theme of the manuscript. DB has helped to examine the phylogenetic aspect of the concept. BGF is responsible for the sample collection and writing of the manuscript. RMK contributed the developing the concept and writing of the manuscript. All the authors contributed to editing the manuscript and approved of its final form.

## Supplementary Material

Additional file 1 Calculation of genetic distance for 3FTx and the PLA_2 _enzymes. The data compares genetic distances among land snakes, sea snakes and sea kraits. Click here for file
