# Supplementary material for: Expression pattern of three-finger toxin and phospholipase A2 genes in the venom glands of two sea snakes, Lapemis curtus and Acalyptophis peronii: comparison of evolution of these toxins in land snakes, sea kraits and sea snakes
Source: BMC Evol Biol. 2007 Sep 27;7:175. doi: 10.1186/1471-2148-7-175 (PMC2174459; doi:10.1186/1471-2148-7-175)
Supplement: Additional file 1 — Calculation of genetic distance for 3FTx and the PLA2 enzymes. The data compares genetic distances among land snakes, sea snakes and sea kraits. [file 1471-2148-7-175-S1.doc]

**A: 3TFx**

| **Species** | **No of**  **sequences** | **Protein sequence Identity**  **(NCBI and Swiss-Prot)** |
| --- | --- | --- |
| *Pseudonaja textilis* | 7 | AAF75221, Q9W7K0, Q9W7J9, Q9W7K2, AAF75223, Q9W7J6, Q9W7J7 |
| *Bungarus multicinctus* | 13 | AAO47841, AAO47839, P79688, Q9W727, CAD56380, CAA06886, CAD56381, Q9YGH9, AAD41806, O12963, Q9YGI8, Q9YGH0, CAD18848 |
| *Bungarus candidus* | 9 | AAL30058, AAL30057, 1JGKA, 1IJCA, P83346, AAL30060, AAL30059, AAL30061, AAT38875 |
| *Enhydrina schistosa* | 2 | P68415, P62389 |
| *Hydrophis cyanocinctus* | 2 | P62376, P25494 |
| *Hydrophis ornatus* | 1 | P68413 |
| *Lapemis curtus* | 3 | P68416, AAL54895, AAL54894, ABN54805 |
| *Aipysurus laevis* | 4 | P19958, P19960, P32879, P19959 |
| *Laticauda colubrina* | 4 | BAA75760, BAA75765, BAA75767, BAA75753 |
| *Laticauda laticaudata* | 5 | BAC78207, BAA75777, BAA75775, BAA75776 |
| *Laticauda semifasciata* | 5 | Q90VW1, CAA35770, P60775, 1NXB, BAA75748 |

**B: PLA_2_**

| **Species** | **No of**  **sequences** | **Protein sequence Identity**  **(NCBI and Swiss-Prot)** |
| --- | --- | --- |
| *Oxyuranus microlepidotus* | 6 | AAZ22642, AAZ22641, AAZ22640, AAZ22643, AAZ22638, AAZ22639 |
| *Oxyuranus scutellatus* | 16 | AAB33760, AAZ22637, AAY47071,  AAY47070, AAZ22635, AAZ22636, AAY47067, P00614, P00615, AAY47066, AAB33759, AAY47069, AAZ22633, AAZ22634, P00616, AAY47068 |
| *Pseudechis porphyriacus* | 8 | P20259, AAZ22668, P20258, AAZ22671, AAZ22669, AAZ22670, AAZ22672, AAZ22667 |
| *Pseudechis australis* | 10 | P20257, P04057, P20250, P20251, P20252, P20254, P20255, P04056, P20256, P20253 |
| *Pseudonaja textilis* | 11 | AAB25359, P23027, AAD40976, Q9W7J4, AAZ22647, AAZ22646, AAZ22648, 1908205C, P23026, AAZ22644, AAZ22645 |
| *Notechis scutatus* | 17 | AAB20783, CAA31125, P08873, P00607, AAZ22649, 2114420A, AAZ22650, 1AE7, P00608, 2NOTA, P00609, AAZ22651, P20146, AAB34122, Q9PSN5, AAZ22652, AAZ22653 |

| *Tropidechis carinatus* | 6 | Q45Z25, Q45Z30, Q45Z29, Q45Z27, AAZ22658, Q45Z28 |
| --- | --- | --- |
| *Austrelaps superbus* | 17 | AAD56564, AAD56563, AAD56562, AAD56561, AAD56560, AAD56559, AAD56558, AAD56557, AAD56556, AAD56555, AAD56554, AAD56553, AAD56552, AAD56551, AAD56550, AAD56410, AAD56409 |
| *Bungarus multicinctus* | 12 | Q9PTA6, Q9PTA5, P00617, Q9PTA1, P59018, P00619, Q9PTA7, Q9PU97, P00618, Q90251, P17934, P00606 |
| *Bungarus candidus* | 8 | AAN16113, AAL30064, AAL30062, BAD06267, AAR07910, BAD06270, AAO84769 |
| *Lapemis curtus* | 3 | AAL55556, Q8UW08, Q8UW31 |
| *Aipysurus laevis* | 1 | P08872 |
| *Aipysurus eydouxii* | 12 | AAT66309, AAT66305, AAT66312, AAT66310, AAT66308. AAT66303, AAT66315. AAT66311, AAT66316, AAT66302, AAT66314, AAT66313 |
| *Laticauda colubrina* | 5 | Q8UUI0, Q8UUH7, Q8UUH9. Q8UUH8, P10116 |
| *Laticauda laticaudata* | 6 | Q8UUI4, Q8UUI3, P19000, Q8UUI1, BAB72251, Q8UUI2 |
| *Laticauda semifasciata* | 10 | Q9I843, Q9I842, Q9I837, Q9I844, Q9I847, P00613, P00612, P00611, Q8JFG2, Q8JFB2 |
